# Supplementary figures and images for: SARS-CoV-2 ORF8 modulates lung inflammation and clinical disease progression
Source: PLoS Pathog. 2024 May 23;20(5):e1011669. doi: 10.1371/journal.ppat.1011669 (PMC11152254; doi:10.1371/journal.ppat.1011669)

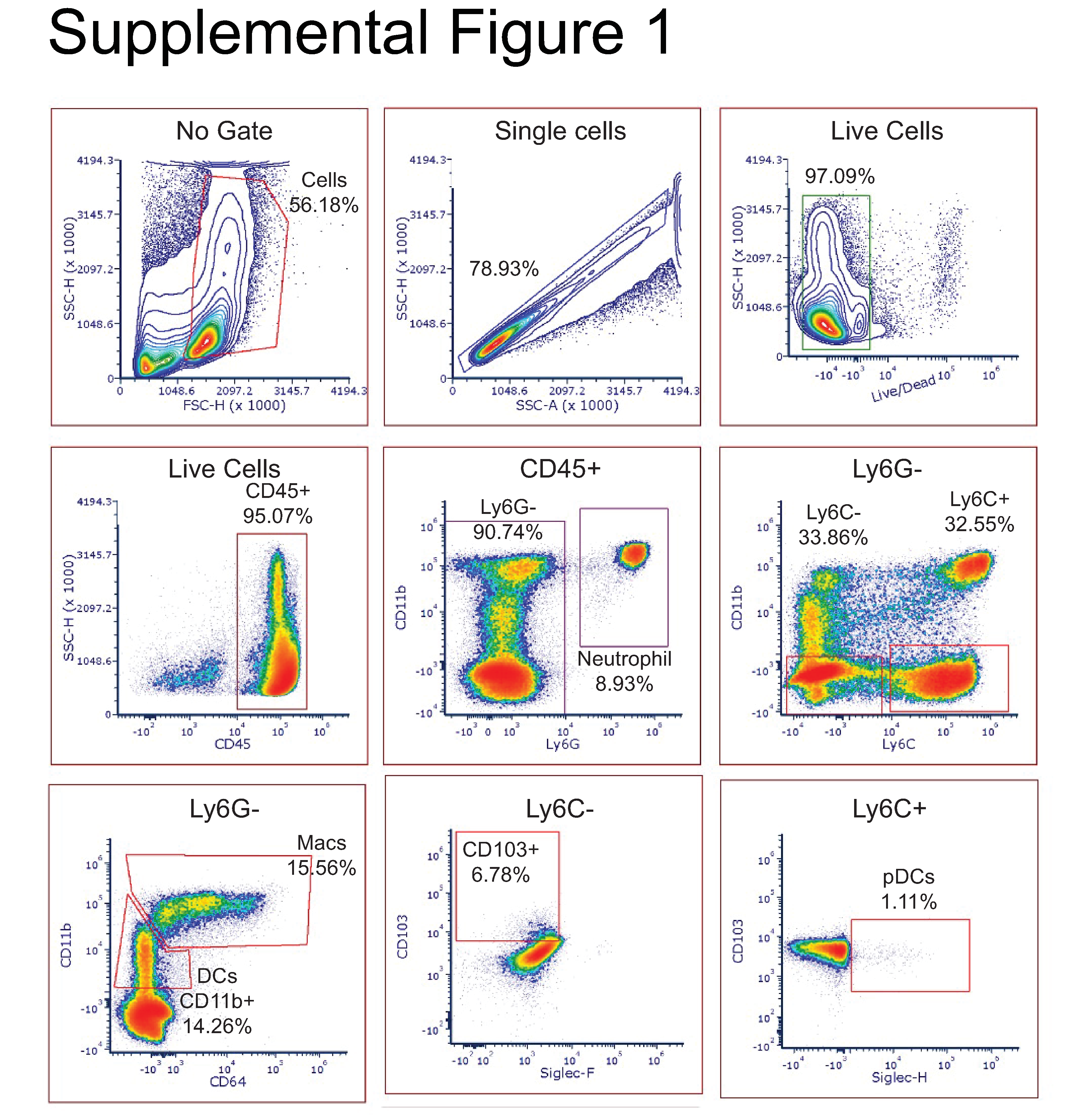

Supplement: S1 Fig — All antibodies are identified in the methods section. The ezhic cell population shown in Fig 4 are macrophages, which for this gating strategy are CD45+/Ly6G-/CD11b+/CD64+. The labels on the axis are the antibodies used in that gating and the label at the top of each box is the population of cells gated from the previous box. (TIF) [file ppat.1011669.s001.tif]
